# Correlation between postpartum depression and the number of births

**DOI:** 10.1192/j.eurpsy.2025.2391

**Published:** 2025-08-26

**Authors:** P. Batchuluun, P. Sarantuya, B. Ganzorig, T. Urtnasan

**Affiliations:** 1 Medical department, Etugen University, Ulaanbaatar, Mongolia

## Abstract

**Introduction:**

Postpartum depression is a disorder that usually occurs six weeks after birth and can last for up to a year. If postpartum depression is not diagnosed and treated, there is a risk of suicide. Postpartum depression affects 18% of mothers worldwide.

**Objectives:**

To investigate the relationship between the prevalence of postpartum depression, the number of births, and postpartum depression.

**Methods:**

Women who gave birth will be surveyed using the Edinburgh test specially prepared by WHO for primary health care practitioners.

**Results:**

41% of the women who gave birth in the study had their first birth, 28% had their second birth, 21% had their third birth, and 10% had four or more births. 2% of women who gave birth had no postpartum depression, 15% had self-managed depression, and 83% had postpartum depression. 4% of women aged 20-24 have no postpartum depression, 20% have self-managed depression, and 76% have postpartum depression. 0% of women aged 25-29 had no postpartum depression, 14.3% had self-managed depression, and 85.7% had postpartum depression. 0% of women aged 30-34 have no postpartum depression, 10% have self-managed depression, and 90% have postpartum depression. 5% of women aged 35-39 have no postpartum depression, 15% have self-managed depression, and 80% have postpartum depression. Among women aged 40-44, 0% had no postpartum depression, 0% had self-managed depression, and 100% had postpartum depression. Among women aged 45-49, 0% had no postpartum depression, 50% had self-managed depression, and 50% had postpartum depression.

Among 41 women with their first child, 2.4% did not have postpartum depression, 17.1% had self-correcting depression, and 80.5% had postpartum depression. Among 28 women with second birth, 0% did not have postpartum depression, 17.9% had self-correcting depression, and 82.1% had postpartum depression. Among 22 women with their third child, 4.5% did not have postpartum depression, 4.5% had self-correcting depression, and 90.9% had postpartum depression. Of the 9 women with four or more births, 0% did not have postpartum depression, 22.2% had self-correcting depression, and 77.8% had postpartum depression.

**Conclusions:**

Postpartum depression is very high among the women who gave birth in the study. According to the results of the study, there is a weak positive correlation between the number of births and the stress of the mother. Stress and insomnia are strongly related. Postpartum depression and insomnia are strongly correlated in Edinburgh. Therefore, there is a need to increase the diagnosis and treatment of postpartum depression.

**Disclosure of Interest:**

None Declared

